# Dengue severity in rheumatoid arthritis patients under treatment with disease‐modifying antirheumatic drugs

**DOI:** 10.1111/1756-185X.15224

**Published:** 2024-06-19

**Authors:** Hsin‐Hua Chen, Chia Hua Chung, Nicole Huang, Theodore F. Tsai, I. Feng Lin

**Affiliations:** ^1^ Taichung Veterans General Hospital Taichung Taiwan; ^2^ National Yang Ming Chiao Tung University Taipei Taiwan; ^3^ Takeda Vaccines, Inc. Cambridge Massachusetts USA

## INTRODUCTION

Immunosuppression associated with disease‐modifying antirheumatic drugs (DMARDs) administered to rheumatoid arthritis (RA) patients can increase risk for infection and more severe outcomes.^1^, ^2^ Few reports are available on the effects of DMARDs on outcomes of dengue, a mosquito‐borne flaviviral infection.^3^, ^4^ Here we report observations on outcomes of dengue, including death, in RA patients treated with various classes of DMARDs.

Dengue is the most common vector‐borne infection globally, with >50 million cases occurring annually in tropical and subtropical locations globally.^5^ Dengue is introduced into the US, Europe, Australia, and other locations by infected travelers, sometimes leading to localized outbreaks. Dengue is the fastest growing vector‐borne infection, with half of the global population estimated to be at risk while, by 2080, that fraction could increase to 60% due to population growth, urbanization, and climate change.^6^

In a previously reported administrative database study, we showed that individuals with various chronic diseases had more severe outcomes of dengue, compared with individuals without those conditions.^7^ Among these co‐morbidities were malignancy, diabetes, chronic heart, renal and lung disease, among others previously shown to be associated with severe dengue; however, we found similarly elevated odds ratios in RA patients, an association not reported previously. We hypothesized that immunosuppressive therapies may have contributed to this elevated risk.

In an extension of the previously published study,^7^ we focused on dengue outcomes in RA patients receiving various classes of DMARDs.

## MATERIALS AND METHODS

### Study design and Dengue patient selection

We conducted a population‐based retrospective cohort study linking laboratory‐confirmed dengue patients in the Notifiable Disease Dataset of Confirmed Cases (NDDCC), Taiwan Center for Disease Control (CDC) to the National Health Insurance Research Database (NHIRD) and the Cause of Death Database using encrypted individuals' identification numbers.^8^, ^9^ Inclusion criteria encompassed confirmed dengue patients who were 18 years or older and were diagnosed between January 1, 2014 and December 31, 2015, during an unusually large dengue outbreak. Those with missing diagnosis dates or identification numbers, multiple reports, or incomplete demographic and socioeconomic information were excluded from the study.

### Exposure and outcome variables

Treatments were classified into four distinct and non‐overlapping groups: biological or target‐synthetic disease‐modifying antirheumatic drugs (b/ts DMARD), conventional‐synthetic disease‐modifying antirheumatic drugs (csDMARD), no DMARDs among dengue‐infected RA patients, and non‐rheumatoid arthritis (RA) dengue patients. Index dates were based on records from NDDCC. For each individual, drug exposure was defined as having received at least one prescription or delivery of medication during the year before the index date of dengue confirmation. Study outcomes were all‐cause mortality, hospitalization, length of stay, and inpatient medical expenditure during the 30‐day follow‐up after the index date. Patient characteristics included age, gender, social economic status (SES) determined by the wages and types of the primary insurance holder, and preexisting noncommunicable diseases (NCDs) before the index date. The NCDs considered in this study included malignancy, RA and related disease, coagulation and hemorrhagic disorders, diabetes, hypertension, coronary artery disease, congestive heart failure, stroke, COPD, asthma, chronic kidney diseases, liver cirrhosis, and major depressive disorder.^7^

### Statistical analysis

Incidence rates were calculated to describe the occurrences of death or hospitalization, while averages were computed for the length of hospital stay (LOS) and inpatient medical expenditure. Multiple logistic regression was employed to estimate the relative odds of 30‐day all‐cause mortality or hospitalization between treatment groups, providing odds ratios (OR) and confidence intervals. Generalized linear models (GLMs) using a gamma distribution and log link function were used to estimate the relative average medical expenditure between treatment groups. Additionally, GLMs with a negative binomial distribution and log link function were used to estimate the relative average LOS between treatment groups. All effects were adjusted for potential confounders including, age, gender, SES, and the number of preexisting NCDs. Covariate‐adjusted survival curves for 30‐day and 1‐year mortality were estimated by multivariable Cox models. All data analyses were conducted using SAS 9.4 and were performed within a secure facility managed by the Health and Welfare Data Science Center (HWDSC) of Taiwan's Ministry of Health and Welfare.

## RESULTS

Among 51 513 laboratory‐confirmed dengue cases, 361 were fatal and 15 052 hospitalized (Table 1). The DMARDs available in Taiwan for health insurance reimbursement in this 2014–15 period were limited to, for csDMARDs: leflunomide, methotrexate, sulfasalazine, and hydroxychloroquine and for b/ts DMARDs: rituximab, etanercept, infliximab, adalimumab, certolizumab pegol, golimumab, opinercept, tocilizumab, abatacept, cyclosporin, azathioprine, ustekinumab, mycophenolic acid, sirolimus, and cyclophosphamide; the latter four drugs are not used in RA‐only patients.

**TABLE 1:** Acute effects of antirheumatic drugs on (A) mortality and incidence of hospitalization and (B) length of hospital stay and inpatient expenditures, among patients with dengue.

(A) Mortality and incidence of hospitalization							
30‐day outcomes	*n*	Number of events	Incidence^a^	Crude incidence ratio^a^	Adjusted odds ratio^b^	95% CI Lower	95% CI Upper
Mortality							
Rheumatoid arthritis							
b/tsDMARD^1^	34	2	5.9	8.39	8.48	1.87	38.56
csDMARD^2^	128	3	2.3	3.34	1.76	0.54	5.78
No DMARD	94	5	5.3	7.59	2.63	0.99	6.98
Non‐rheumatoid arthritis	51 513	361	0.7	1	(Reference)		
Hospitalization							
Rheumatoid arthritis							
b/tsDMARD	34	16	47.1	1.61	1.53	0.76	3.04
csDMARD	128	60	46.9	1.6	1.35	0.94	1.93
No DMARD	94	48	51.1	1.75	1.39	0.91	2.11
Non‐rheumatoid arthritis	51 513	15 052	29.2	1	(Reference)		

(B) Inpatient expenditures and length of hospital stay						
30‐day outcomes	*n*	Mean	Crude mean ratio^c^	Adjusted mean ratio^b^	95% CI Lower	95% CI Upper
Inpatient expenditures						
Rheumatoid arthritis						
b/tsDMARD	34	898.28	3.23	1.63	0.63	4.23
csDMARD	128	878.73	3.16	2.58	1.57	4.22
No DMARD	94	566.02	2.04	0.88	0.49	1.56
Non‐rheumatoid arthritis	51 513	277.98	1	(Reference)		
Length of hospital stay, days						
Rheumatoid arthritis						
b/tsDMARD	34	3.97	1.99	1.23	0.51	2.98
csDMARD	128	4.05	2.04	1.44	0.91	2.27
No DMARD	94	3.23	1.62	0.95	0.56	1.62
Non‐rheumatoid arthritis	51 513	1.99	1	(Reference)		

Abbreviations: b/tsDMARD, biological/target‐synthetic disease‐modifying antirheumatic drug; csDMARD, conventional‐synthetic disease‐modifying antirheumatic drug; DMARD, disease‐modifying antirheumatic drug.

^a^ Incidence: number of events per 100 persons; the ratio of incidence to that of the reference group.

^b^ Adjusted for age, sex, socioeconomic status, year of diagnosis, and number of co‐morbidities.

^c^ Ratio of average expenditure or length of hospital stay in days to that of the reference group.

^1^ b/tsDMARDs—The b/ts DMARD therapies comprised rituximab, etanercept, infliximab, adalimumab, certolizumab pegol, golimumab, opinercept, tocilizumab, abatacept, ciclosporin, azathioprine, ustekinumab, mycophenolic acid, sirolimus, and cyclophosphamide; the latter four drugs are not used in RA‐only patients.

^2^ csDMARDs—The csDMARD therapies included leflunomide, methotrexate, sulfasalazine, and hydroxychloroquine.

**FIGURE 1:**
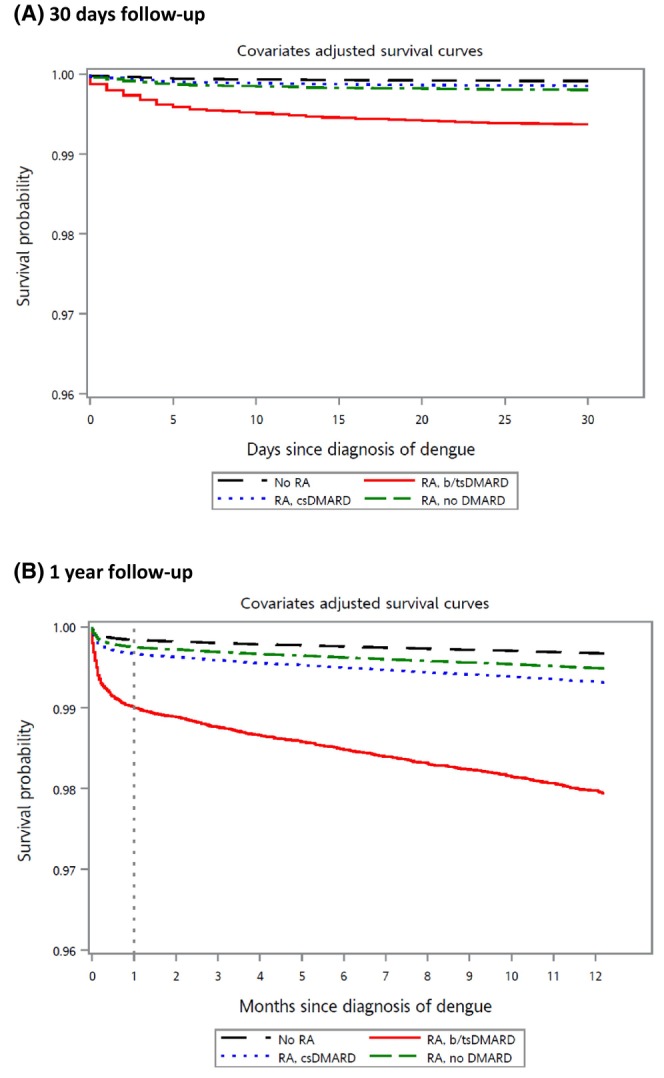
Covariate‐adjusted survival estimates of death at (A) 30 days and (B) 1 year of follow‐up since diagnosis of dengue. The graphs show the data on an enlarged *y* axis considering low mortality rates. *p*‐Value = .005 for comparison between the RA‐t/bs DMARD and non‐RA groups in 30 days follow‐up. A: 30 days follow‐up. B: 1 year follow‐up.

We found that in the 30‐day period following dengue diagnosis, RA patients treated with b/tsDMARDs, compared with non‐RA patients, were more likely to die, with an adjusted OR of 8.48 (95% CI 1.87–38.56) (Table 1). The broad confidence intervals reflect the relatively small number of deaths in the various groups, but there was a suggestion of higher mortality in the other RA patient groups. Deaths in b/tsDMARD recipients occurred earlier than in other groups and the difference in survival progressed over 1 year (Figure 1).

Hospitalizations of RA patients treated with csDMARDs or under no treatment trended higher; for patients on csDMARDS, their LOS and inpatient costs were or trended toward being higher.

## DISCUSSION

Dengue is an *Aedes aegypti*‐borne flaviviral infection that typically results in a self‐limited illness of short duration, with a fatality rate of <0.1%.^10^ Infection with any of the four antigenically distinct viral serotypes, Dengue 1–4, is believed to provide life‐long immunity against viruses of that serotype and temporary cross‐protection against infection, with the other three lasting ~18 months. However, after that interval, a second infection can produce an exacerbated illness characterized by rapid hemodynamic changes due to plasma leakage across endothelia, enhanced antibody‐dependent enhanced viral replication, and a pathophysiological host inflammatory response. Advanced age and co‐morbidities are risk factors for severe outcomes that may occur in primary infections.^11^ While diabetes, hypertension and chronic heart, lung, and kidney diseases have been shown consistently to contribute to severe outcomes of acute dengue, unexpectedly, we identified RA as another co‐morbidity‐risk factor, with a fourfold higher adjusted odds ratio for death within 30 days of dengue diagnosis, compared with patients without RA.^7^ We hypothesized that immunosuppressive therapy was behind this increased risk. Here we report that only RA patients treated with b/ts DMARDs experienced an 8.48 higher adjusted odds for death from acute dengue, compared with dengue patients without RA, although confidence intervals were broad. RA patients treated with csDMARDs trended toward being more likely to be hospitalized and with a longer, more costly stay but were not more likely to die, compared with non‐RA patients. RA patients on no DMARDs were not distinguishable from non‐RA dengue patients for these outcomes.

These observations suggest differences in the immunosuppressive potency or specificity of b/ts DMARDs compared with csDMARDs in their impairment of responses to dengue infection. We speculate that in the first case, residual host immune responses could not overcome infection while in the second, immunosuppression‐impaired host responses led to a more severe and protracted but, ultimately, survivable course (Figure 1).

These conclusions may not be generalizable to other locations where dengue is endemic and most adults have experienced one or more infections. Dengue is not endemic in Taiwan although the virus frequently is introduced, due to the island's proximity to endemic locations in Southeast Asia. Outbreaks are relatively small and are confined geographically almost exclusively to the southern half of the island where *Ae. aegypti* is present. *Ae. albopictus* is broadly distributed but because it is comparatively inefficient as a vector, few case clusters occur in the northern half of the island where it is the sole vector species; overall, transmission occurs at such a low and sporadic level that population seroprevalence on the island is <~5%.^12^

The clinical course and outcomes of dengue in Taiwan reflect principally features of dengue‐naïve individuals. In contrast, where dengue is endemic, repeated lifetime infection provides broad immunity to infection in adults. When as adults, they receive immunosuppressive therapies, unaffected residual immunity may provide protection against severe disease, as noted for influenza.^13^ Furthermore, outcomes of dengue infection in people living with HIV infection under treatment and in solid organ transplant patients in the dengue endemic setting do not appear to experience exacerbated infections.^14^, ^15^ Studies of b/ts DMARD therapy of RA patients in locations where dengue is endemic are needed to confirm this conjecture.

The observations here may apply to dengue‐naive travelers from Europe, the United States, and other locations where dengue is not endemic. RA patient travelers receiving DMARDS, especially b/tsDMARDs, may potentially be at risk for severe outcomes if exposed to dengue during travel and should be counseled accordingly. Outcomes of dengue in patients receiving DMARDs for other clinical conditions and responses of DMARD‐treated patients to dengue vaccines should be examined.^13^

## CONCLUSION

DMARD‐treated RA patients who are dengue‐naïve and who may be exposed, for example, during travel should be counseled that they may be at increased risk for severe outcomes, especially those receiving b/ts DMARDs.

## AUTHOR CONTRIBUTIONS

TFT, NH, IFL, and HHC contributed equally to the conceptualization, analysis, interpretation, and writing of the study and its results. CHC and IFL were principally responsible for designing the analysis and data interpretation.

## CONFLICT OF INTEREST STATEMENT

Theodore F Tsai MD MPH FIDSA is a Vice President, Immunization Science and Policy and full‐time employee of and shareholder in Takeda Vaccines, a manufacturer of a dengue vaccine.

## Data Availability

The data that support the findings of this study are available from Ministry of Health, Taiwan. Restrictions apply to the availability of these data, which were used under license for this study. Data are available from (https://dep.mohw.gov.tw/DOS/cp‐5119‐59201‐113.html) with the permission of Ministry of Health, Taiwan.
